# Myxedema Crisis Presenting with Seizures: A Rare Life-Threatening Presentation—A Case Report and Review of the Literature

**DOI:** 10.1155/2017/4285457

**Published:** 2017-05-02

**Authors:** Sonali Sihindi Chapa Gunatilake, Uditha Bulugahapitiya

**Affiliations:** Endocrinology, Colombo South Teaching Hospital, Kalubowila, Sri Lanka

## Abstract

Myxedema crisis is a life-threatening extreme form of hypothyroidism with a high mortality rate if left untreated. Myxedema crisis is commonly seen in older patients, especially in women, and is associated with signs of hypothyroidism, hypothermia, hyponatraemia, hypercarbia, and hypoxemia. Patients might present with different organ specific symptoms. Seizures are a recognized but rare manifestation of myxedema with a very high mortality rate. Prompt diagnosis and appropriate management may improve the prognosis. Many contributory factors may involve development of seizures in a patient with myxedema. Hyponatraemia is one such cause, which is seen in moderate-severe form in the background of myxedema. We report an elderly male who presented with generalized tonic clonic seizure preceded by memory impairment and drowsiness. He had moderate hyponatraemia and very high thyroid stimulatory hormone levels in association with low free thyroxin levels. Diagnosis of myxedema crisis was made and patient was successfully treated with sodium correction and thyroid hormone replacement.

## 1. Case Presentation

A 68-year-old male patient was brought to the Emergency Treatment Unit with first episode of generalized tonic clonic seizure, which lasted for 15 minutes.

Detailed history revealed that he was having mild memory impairment and drowsiness for the past 1 month prior to the index admission. There was no associated fever, diarrheal illness, respiratory symptoms, morning headache with vomiting, or focal neurological deficit prior to the development of fits. There was no history of trauma to head. He did not have any chronic illness or fits in the past, did not undergo any surgeries, and was not on any medications. There was no family history of cardiovascular events or epilepsy. He is a nonsmoker and has not consumed alcohol. He was not an illicit drug abuser.

Following admission, patient remained drowsy with only a mild improvement of conscious level following the seizure.

On examination, his body mass index was 27 kg/m^2^ (height, 1.65 cm; weight, 73.5 kg). He had a puffy face with significant periorbital swelling and bilateral nonpitting ankle edema. His skin was dry and coarse. Neck examination revealed no lymphadenopathy or goiter. His body temperature was 36°C. Vital parameters revealed a heart rate of 45 beats/min, blood pressure of 140/100 mmHg, and a respiratory rate of 12 cycles/min with an oxygen saturation of 94% on air. Glasgow coma scale (GCS) was 10/15 on admission which had improved to 12/15 with persisting drowsiness. He did not have any evidence of external injuries. There was no neck stiffness or detectable focal limb weakness. His ankle jerk was slow relaxing, planta response was flexor, and his fundi were normal. Examination of the respiratory system and abdomen was normal.

Following the clinical evaluation, meningoencephalitis, intracranial space occupying lesion, myxedema, metabolic encephalopathy, and toxin induced disease were taken as differential diagnoses. Preceding memory disturbances, facial puffiness, dry skin, hypothermia, bradycardia, low respiratory rate, and slow relaxing reflexes were supportive of the diagnosis of myxedema.

Basic investigations revealed, haemoglobin, 10.5 g/dL, with macrocytosis, normal white cell count, and normal inflammatory markers. His random blood sugar was 85 mg/dL, liver profile revealed AST of 50 U/L (<20), ALT of 65 U/L (<17), and serum creatinine of 1.3 mg/dL (0.8–1.2). Noncontrast computed tomography of the brain was normal excluding the possibility of intracranial lesion. Electroencephalogram revealed diffuse slow waves and was suggestive of metabolic encephalopathy. Electrocardiogram showed sinus bradycardia with small QRS complexes. ST segments were depressed and T waves showed inverted pattern in all the leads. Echocardiogram showed a mild-to-moderate amount of pericardial effusion with good left ventricular functions but had no evidence of cardiac tamponade. In addition, his creatinine kinase (CK) value was 455 U/L (24–195). Septic screening was negative.

His serum sodium level (Na^+^) was 125 mmol/L and potassium was 4.0 mmol/L. Further evaluation revealed a low serum osmolality (260 mOsm/L) with a urinary osmolality of 426 mOsm/L and urinary sodium excretion of 54 mmol/L. His random cortisol level prior to initiating treatment was 560 nmol/L and thyroid stimulating hormone (TSH) and free thyroxin level (fT4) were >100 mU/L (0.4–4) and 0.32 ng/dL (0.9–1.7), respectively. Lumbar puncture and cerebrospinal fluid analysis was performed to exclude the possibility of meningoencephalitis and CSF results were normal.

Diagnosis of myxedema was made on clinical as well as biochemical evidence. In addition to the very high TSH and low fT4 levels, patient had macrocytic anaemia, mild pericardial effusion on echocardiography, hyponatraemia in the background of normal hydration status, elevated liver enzymes, and high CK value in support of the above diagnosis. It was further supported by the high total cholesterol level of 310 mg/dL (<200 mg/dL) found on subsequent evaluation. A definitive precipitation factor was not identified in our patient.

As the possible causes for the presentation with fits and persistent drowsiness, hyponatraemia and/or myxedema were considered. Our patient had moderate degree of hyponatraemia (125–129 mmol/L). Although overt neurological symptoms are seen in severe hyponatraemia (<125 mmol/L), especially when the Na^+^ < 115 mmol/L [1], as the patient was having persistent drowsiness, he was initially managed with Na^+^ correction. He was given one bolus of 3% NaCl 100 ml over 20 min on admission following which his GCS had improved to 13/15. Thereafter, hyponatraemia was managed with fluid restriction. After 4 hours, serum Na^+^ was 128 mmol/L. In addition, general supportive measures including gradual rewarming were initiated.

Patient was commenced on intravenous (IV) glucocorticoids (hydrocortisone 50 mg 6 hourly) after taking a blood sample for random cortisol and treatment was continued until glucocorticoid deficiency was ruled out. After initiating glucocorticoids, he was treated with oral levothyroxine 400 *μ*g initial dose via nasogastric tube followed by oral levothyroxine 100 *μ*g daily. Oral form was used instead of recommended IV form due to the unavailability of intravenous levothyroxine. Recommended dose is IV levothyroxine 200–400 *μ*g followed by 1.6 *μ*g/kg replacement dose, where 75% of it is given if the daily replacement is done with IV levothyroxine [2]. A lower dose was used in our patient after the initial dose (calculated dose is 1.6 *μ*g/Kg × 80 Kg = 128 *μ*g/day) as he was elderly and to prevent any cardiovascular morbidity.

**Figure 1:**
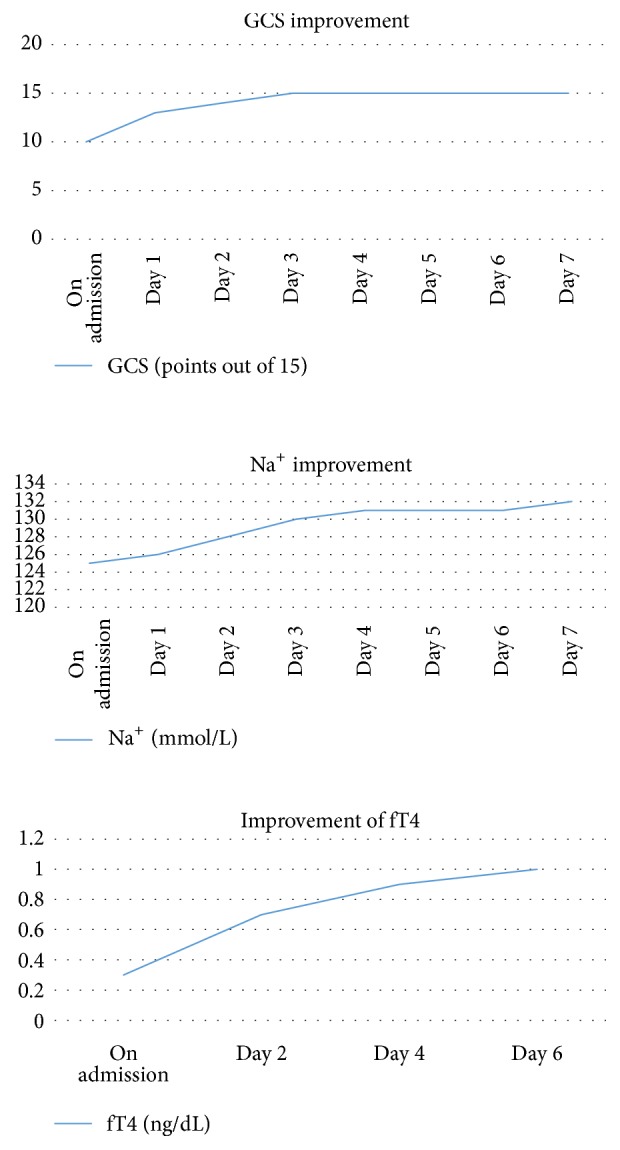
Improvement of parameters following treatment.

Careful monitoring was done with regard to clinical improvement, serum Na^+^ level daily, and fT4 every 2 days as in Figure 1.

Following good clinical recovery, he was discharged and reviewed in six weeks. His fT4 was 1.12 ng/dL and TSH was 10.4 mU/L. Slow titration was done in order to achieve normal TSH range. His memory and cognition had markedly improved with resolution of facial puffiness. Biochemical parameters including Na^+^, liver enzymes, serum creatinine, CK, red cell indices, and echocardiogram had also normalized at 3 months of follow-up.

## 2. Discussion

Myxedema crisis/coma is a rare life-threatening clinical condition that represents severe hypothyroidism with physiological decompensation [3]. The term myxedema coma is a misnomer, and myxedema crisis may be an appropriate term as quite a few patients are obtunded, rather than frankly comatose [4]. It is rare and unrecognized. Exact prevalence of myxedema coma is unknown. Even with early detection and appropriate treatment, mortality ranges from 30 to 60% where most die due to respiratory failure, sepsis, and gastrointestinal bleeding [5, 6]. Myxedema crises occur mostly in persons 60 years or older and nearly 80% of cases occur in females [7]. However, myxedema coma occurs in younger patients as well, with more than 30 documented cases of pregnant women [8].

Low intracellular triiodothyronine (T3) secondary to hypothyroidism is the basic underlying pathology in myxedema crisis which leads to hypothermia and suppression of cardiac activity. The body tries to compensate by neurovascular adaptations including chronic peripheral vasoconstriction, mild diastolic hypertension, and diminished blood volume. Decreased central nervous system sensitivity to hypoxia and hypercapnia leads to respiratory failure. Altered vascular permeability leads to effusions and anasarca. Water retention and hyponatraemia occurs secondary to reduced glomerular filtration rate, decreased delivery to the distal nephron, and excess vasopressin [9]. Decreased gluconeogenesis, precipitating factors like sepsis and concomitant adrenal insufficiency, may contribute to hypoglycemia. In addition to the generalized depression of cerebral function, hyponatraemia, hypoglycemia, hypoxemia, and reduced cerebral blood flow can precipitate focal or generalized seizures and worsen the level of consciousness as in the index case.

Most of the patients with myxedema crisis have primary hypothyroidism and secondary hypothyroidism account to 5% of the cases [10]. Dutta et al. reported that 39% of patients present with myxedema crisis had hypothyroidism detected only at the time of crisis [11] as in our patient.

Clinical presentation may vary but almost all patients have altered mentation and 80% have hypothermia. In addition to the characteristic features of hypothyroidism, patients may present with some atypical features as heart blocks, prolonged QT interval and arrhythmias, myocardial infarction, pericardial/pleural effusions, respiratory depression, hypercapnia, bleeding manifestations with prolonged APTT, and acquired von Willebrand factor defects and psychosis [12, 13]. Neurological manifestations in myxedema may range from alteration of mental status with slowness, decreased concentration and lethargy, headache, cranial nerve palsies, hoarseness, myopathy, neuropathy, reflex changes, ataxia, psychotic episodes, and fits. Ultimate result would be a coma state and role of hypothermia, CO_2_ narcosis, cerebral edema, and other metabolic disturbances in the genesis of coma should be looked into.

Occurrence of seizures in myxedema can have several mechanisms but myxedema itself can precipitate seizure activity. The cause of epileptic seizure activity in hypothyroidism is unknown. It may be due to cerebral oedema secondary to expansion of the extracellular fluid volume [14]. This may be related to inappropriate antidiuretic hormone (ADH) secretion and hyponatraemia or hypoventilation with postanoxic encephalopathy, which can further precipitate seizure activity.

Hyponatraemia is reported in up to 10% of hypothyroid patients, although it is usually mild and rarely causes symptoms [15]. In water-loading studies, patients with hypothyroidism have a diminished ability to excrete free water and fail to achieve maximum urine dilution. Although some studies have reported elevated ADH levels in patients with hypothyroidism the literature is inconsistent [15]. The reduction in cardiac output and glomerular filtration rate observed in severe hypothyroidism may be a nonosmotic stimuli to ADH release. However, recent data suggests that the hypothyroidism induced hyponatraemia is rather rare and probably occurs only in severe hypothyroidism and myxedema [16]. Patients with a low plasma sodium had a lower mean Free T4 concentration and higher mean TSH concentration than Free T4 concentration and mean TSH concentration of the patients with a normal plasma sodium concentration. Treatment of hypothyroidism and fluid restriction are usually adequate for the management of mild hyponatraemia in hypothyroidism. Patients with possible hyponatraemic encephalopathy should be urgently treated according to the protocols of management of severe hyponatraemia but caution must be taken to avoid rapid correction of chronic hyponatraemia, which might put patients at risk for central pontine myelinolysis.

Patient in the index case presented with a generalized tonic clonic seizure in the background of newly diagnosed severe hypothyroidism and moderate hyponatraemia, which is relatively rarely reported in the literature in association with myxedema and fits. Both factors could have contributed for the development of seizures although classically Na^+^ level of <120 mmol/L is known to cause seizures. He was initially managed with 3% NaCl as he was having a lower level of GCS on admission in the background of fits followed by persistent drowsiness.

Management of myxedema crisis involves replacement of thyroxin hormones with additional supportive care. Prior to thyroxin replacement, glucocorticoid replacement should be considered as the clinical features of myxedema crisis and cortisol deficiency may overlap; hence thyroid hormone replacement may increase cortisol clearance and may aggravate cortisol deficiency. In addition, precipitant cause should be sought and treated.

Thyroxin replacement is recommended in the form of intravenous (IV) tetraiodothyronine (T4), mainly to avoid poor gastrointestinal absorption. T4 therapy provides a smooth, steady, and slow onset of action with relatively lesser number of adverse events [4]. T4 therapy avoids major peaks and troughs in body. Values of serum T4 may be easy to interpret. However, triiodothyronine (T3) is the active hormone in the body, and in a setting of severe illness there may be a decreased conversion of T4 to T3. Advantages of using T3 include a rapid onset of action, an earlier beneficial effect on neuropsychiatric symptoms, and significant clinical improvement within 24 hours. Several options are available for the treatment of myxedema [2]:

1. IV T4 loading dose of 200–400 *μ*g bolus (to replenish body stores) followed by 75% of the calculated dose of [1.6 *μ*g/Kg × 75%] IV T4 per day till patient is alert to take oral thyroxin
2. IV T3 10–20 *μ*g followed by 2.5–10 *μ*g every 8 hours during first 2 days till patient is alert to take oral thyroxin
3. Combination of IV T4 4 *μ*g/Kg (or 200–300 *μ*g) + IV T3 10 *μ*g bolus followed by T4 100 *μ*g in 24 hours and 50 *μ*g/day thereafter with T3 2.5–10 *μ*g every 8 hours till patient recovers

Although there are beneficial effects, poor availability, fluctuations in serum levels of T3, adverse cardiac effects, and limited availability may limit the use of IV T3. There is a controversy on the ideal modality of treatment and American Thyroid Association recommends combination of IV T4 and T3 [2]. Measurement of thyroid hormones every 1-2 days is suggested. Yamamoto et al. reported that doses of LT4 more than 500 *μ*g per day and LT3 more than 75 *μ*g/day were associated with increased mortality [17].

Oral administration of T4 through nasogastric tube has proved to be equally effective with a drawback that gastric atony may prevent absorption and put the patient at risk for aspiration. Dutta and colleagues compared 500 *μ*g of oral loading dose of T4 with 150 *μ*g of maintenance dose orally and 200 *μ*g of T4 intravenously followed by 100 *μ*g T4 intravenously until they regained their vital functions and were able to take oral medications in patients with myxedema crisis and did not find any difference in outcome among the patients [11]. Arlot et al. reported that oral absorption of T4 is variable, but clinical response occurs quickly even in myxoedema ileus after comparing oral T4 500 *μ*g stat dose followed by 100 *μ*g/day with IV T4 in patients with myxedema [6]. But all above studies had used higher doses of oral T4 compared to the IV T4 dose. A lower initial dose of T4 should be administered to patients who are frail or have other comorbidities, particularly cardiovascular disease. Thyroid hormones may be measured every 1 to 2 days to identify the response. We used oral T4 for our patient who had shown marked improvement clinically as well as biochemically over 1 week.

## Abbreviations

ADH: Antidiuretic hormone
ALT: Alanine transaminase
APTT: Activated partial thrombin time
AST: Aspartate transaminase
CK: Creatinine kinase
CSF: Cerebrospinal fluid
CT: Computed tomogram
GCS: Glasgow coma scale
IV: Intravenous
NaCl: Sodium chloride
TSH: Thyroid stimulating hormone
fT4: Free tetraiodothyronine
fT3: Free triiodothyronine.
